# Changes in abdominal obesity in Chilean university students stratified by body mass index

**DOI:** 10.1186/s12889-015-2587-3

**Published:** 2016-01-13

**Authors:** Marco Cossio-Bolaños, Catalina Vilchez-Avaca, Victor Contreras-Mellado, Cynthia Lee Andruske, Rossana Gómez-Campos

**Affiliations:** 1 Department of Physical Activity Sciences, Catholic University of Maule, Talca, Chile; 2 Faculty of Physical Education, State University of Campinas, São Paulo, Brazil; 3 Red Iberoamericana Biological Research in Human Development, Arequipa, Peru; 4 Program of sports and physical activity, Universidad de Talca, Talca, Chile; 5 Master of Science in Physical Activity, Catholic University of Maule, Talca, Chile; 6 Universidad Autonoma de Chile, Talca, Chile; 7 Universidad Científica del Sur, Lima, Peru; 8 Av. San Miguel s/n., Talca, Chile

## Abstract

**Background:**

Studies based on Body Mass Index (BMI) and waist circumference (WC) are generally used to examine the prevalence and tendency of overweight and obesity. These studies help determine the socioeconomic development of a country and improve public health policies. Therefore, the goal of this research was to determine the trend of change in abdominal obesity of Chilean university students according to the Body Mass Index (BMI) measured in intervals of three and six years.

**Methods:**

For this study, a total of 1598 students of both sexes ranging in age from 18 to 26 from a Chilean university were evaluated. Students were assessed commencing in 2007 (372 males and 315 females), 2010 (250 males and 330 females), and ending in 2013 (153 males and 178 females). During the three transversal assessments, weight, height, and waist circumference were evaluated. BMI was calculated for both sexes.

**Results:**

No significant differences were found in age and BMI during the three years evaluated (2007, 2010, and 2013). In 2013, waist circumference (WC) increased significantly (*p* < 0.001 for both sexes). Moreover, in 2013, in all the percentiles evaluated, high values of WC were compared in relation to previous years. Furthermore, in 2013, in all four BMI categories (underweight, normal, overweight, and obese), the university students showed significant increases in WC (Females: *p* = 0.004; Males: *p* = 0.035) whereas in 2007 and 2010, the values remained relatively stable.

**Conclusions:**

BMI remained constant during 2007, 2010, and 2013. However, the university students of both sexes showed greater risk of abdominal obesity as a result of increased WC in 2013.

## Background

Student trends have become important work for human biological researchers to determine the socioeconomic development of a country for improving public health policies [1]. A secular trend is defined as the differences between individuals or groups of the same age and sex associated with the year or decade of birth [2]. It has become an important biological indicator in terms of health. Furthermore, these types of studies possess a fundamental characteristic that allows one to infer the increase of the prevalence of diseases associated with obesity, hypertension, glucose intolerance, and dyslipidemia, not only the early type found in childhood and adolescence but also during adulthood.

A number of studies have established various criteria to determine secular trends in children, adolescents, and adults [3–5]. Currently, researchers have directed their attention to studying the prevalence and tendency of overweight and obesity. These studies are based on Body Mass Index (BMI) and waist circumference (WC) [6], generally used for studying diverse populations worldwide [7].

In general, during the last decades, it has been widely acknowledged that height has tended to stabilize primarily in the developed countries. However, weight continues to increase resulting in forms of obesity [8] and increasing with greater frequency worldwide [7, 9]. In fact, various countries in South America are in the process of nutritional transition. Therefore, it is expected that these societies experience important changes in their demographics, in their diets, and in their respective lifestyles.

In essence, Chile is not far behind in this trend. In the past few years, a number of studies have shown an increased prevalence for overweight, obesity, and metabolic risk in young university students [10–13]. This excessive increase is related to the rapid socio-economic advances seen in the last decades [14, 15]. Therefore, the assumption is that young university students studying probably demonstrate significant changes in body fat in short periods of time. Therefore, the objective of this study was to determine the trend in changes in abdominal obesity in Chilean university students in relation to the BMI in measured intervals of three and six years.

## Methods

### Study design, sample and subjects

The research design was cross-sectional. In this study, young Chilean university students from the University of Talca (UTAL), Lircay Campus UTAL (Talca, Chile) answered three transversal questionnaires in 2007, 2010, and 2013. UTAL is a Chilean state university. It is located northeast of the city of Talca. It is 270 km south of Santiago (the capital of Chile). The economy of Talca is based primarily on agriculture and cattle while the wine industry plays a significant role in the local economy.

For the three years of this study, non-probabilistic sampling (quotas) was used. The data collected from the first survey included responses from 687 university students (372 males and 315 females); for the second survey, 580 (250 males and 330 females); and for the third survey, 331 (153 males and 178 females) for a total of 1598 young university students. Furthermore, during all three years, subjects dropped out of the study. The attrition rate ranged from 12.7-to-15.6 % for males and 18.3 % for females. Age ranged from 18 to 26 years. Students below and above the established age range, those not providing informed consent, or students not attending the day of the evaluation were excluded from the research. Students were recruited from their respective faculties and invited to participate voluntarily.

Collection of the anthropometric information was approved by the Ethics Committee of the University of Talca (UTAL). All of the subjects provided informed consent before anthropometric information was collected from them.

### Anthropometric measurements

All anthropometric data was collected at the beginning of each academic year of the study (March-April). The technical personnel were trained prior to collecting data in order to become familiar with the standardized evaluation measurement protocol for collecting anthropometric data. Each research year, 10 % of the sample was evaluated twice. The technical measurement error (TME) was less than 2 % for the three evaluation years. In effect, the protocol described by the International Society for the Advancement of Kinanthropometry (ISAK) [16] was used as the basis for the study. Weight (kg) was measured with a Tanita digital (United Kingdom, Ltd.) scale with a precision of 100 g and a scale of 0 to 50 kg. Height (cm) was measured with an aluminum Seca estadiometer (Seca Gmbh & Co. KG, Hamburg, Germany) graduated in millimeters with a scale of 0 to 250 cm. The circumference of the waist (cm) was measured at the mid-point between the lower ribs and the top of the iliac crest with a metal belt Seca measuring tape in millimeters with a precision of 0.1 cm.

The BMI (kg/m^2^) was used to classify the young university students into categories of Underweight (BMI < 18.5 kg/m^2^), Normal (18.5 kg/m^2^ ≤ BMI < 25 kg/m^2^), Overweight (25 kg/m^2^ ≤ BMI < 30 kg/m^2^), and Obese (BMI ≥ 30 kg/m^2^) [17].

### Statistical analysis

The Kolmogorov-Smirnov (K-S) test was run on all the variables. It was conducted separately for each sex and evaluation year in order to determine the normal distribution. The data was analyzed using descriptive statistics in order to find the frequencies and percentages for the variable categories. Means and standard deviation (SD) were determined for the continuous variables. In addition, percentile distribution was determined: p5, p15, p50, p85, and p95.

The differences between the three evaluation years and the differences between the four BMI categories were verified by one-way ANOVA. Post hoc Tukey test was used to identify points of difference. Differences between percentiles were verified through fraction 100 log (percentile from 2007/2010, 2007/2013, 2010/2013). SPSS 16 (SPSS Inc., Chicago, IL, USA) wias used for the statistical analysis, adopting a level of significance of 5 %.

## Results

Table 1 below shows the characteristics and evaluations of the students enrolled in the university during the 3 year project.

**Table 1:** Characteristics of the sample studied

		Males		Females	
	Total	*n*	%	*n*	%
Age distribution					
<20 years	826	376	23.5	450	28.2
21–23 years	641	320	20	321	20.1
>24 years	131	82	5.1	49	3.1
Total	1598	778	48.6	820	51.4
Year of data collection					
2007	687	373	23.3	314	19.7
2010	580	252	15.8	328	20.5
2013	331	153	9.6	178	11.1
Total	1598	778	48.7	820	51.3

*%* percentage, *n* sample

Table 2 shows the means and ± SD for age, weight, height, and WC for both sexes of the university students. No significant differences were found in age and BMI during 2007, 2010, and 2013. The mean values remained relatively stable in 2007 and 2010. However, during the 2013 evaluation, both sexes showed significant increases in body weight and WC when compared with the 2007 and 2010 evaluations. With regard to the prevalence of overweight and obesity, values increased in both sexes (4.1 % for males and 6.8 % for females) throughout 2007 to 2013. Only two categories, overweight and obese, were considered since an abnormal accumulation of fat may cause health problems.

**Table 2:** Mean and SD values for age and anthropometric characteristics and prevalence of overweight and obesity in young university students

Variables		Males			Females		
	Years	*n*	X	SD	*n*	X	SD
Age (years)	2007	373	21.0	1.8	314	20.5	1.6
	2010	252	20.8	1.8	328	20.6	1.7
	2013	153	20.9	2.2	178	20.7	2.0
	Total	778	20.9	1.9	820	20.6	1.7
Weight (kg)	2007	373	72.4	11.3	314	59.6	8.5
	2010	252	72.5	10.5	328	60.1	10.5
	2013	153	74.5	12.6^ab^	178	62	10.5^ab^
	Total	778	72.9	11.3	820	60.3	9.8
Height (cm)	2007	373	172.7	6.7	314	161.1	6.0
	2010	252	174.4	5.8	328	161.7	6.0
	2013	153	173.6	6.4	178	161.1	5.7
	Total	778	173.4	6.4	820	161.3	5.9
Waist Circumference (cm)	2007	373	83.9	9.2	314	76.0	7.7
	2010	252	83.8	7.9	328	75.8	9.1
	2013	153	89.4	10.0^ab^	178	83.4	10.5^ab^
	Total	778	84.6	9.3	820	77.6	9.4
BMI (kg/m^2^)	2007	373	24,3	3,2	314	22,9	2,9
	2010	252	23,8	3	328	23	3,5
	2013	153	24.6	3.8	178	23.9	3.6
	Total	778	24.2	3.3	820	23.1	3.3
			Overweight	Obesity		Overweight	Obesity
Prevalence BMI (BMI categories)	2007	373	96 (25.7 %)	19 (5.1 %)	314	57 (18.2 %)	7 (2.2 %)
	2010	252	66 (26.2 %)	15 (6.0 %)	328	42 (12.8 %)	19 (5.8 %)
	2013	153	49 (32.0 %)	14 (9.2 %)	178	38 (21.3 %)	16 (9.0 %)
	Total	778	211 (27.1 %)	48 (6.2 %)	820	137 (16.7 %)	42 (5.1 %)

*X* means, *SD* Standard deviation, *BMI* Body mass index

^a^significant difference in relation to 2007 (*p* < 0.05); ^b^significant difference in relation to 2010 (*p* < 0.05)

Table 3 below illustrates the comparisons of BMI and WC based on percentile distributions. For both sexes, the BMI values were relatively similar in percentiles p5, p15, and p50. However, for 2013, the increase was relatively significant when percentiles p85 and p95 were compared to the two previous years. In this sense, a slight positive increase in BMI occurred in percentiles p85 and p95. These increases amounted to 2.8–4.1 cm in males and 2.7–5.9 cm in females.

**Table 3:** Comparison of the body fat (BMI and WC) of young university students of both sexes by percentile during 2007, 2010, and 2013

Years	*n*		BMI (kg/m^2^)					WC (cm)			
		Centiles									
		5	15	50	85	95	5	15	50	85	95
Males											
2007	373	20.0	21.0	24.0	27.6	30.9	71.0	75.0	83.0	90.0	100.1
2010	252	20.0	21.0	23.7	26.8	29.5	71.0	75.0	82.0	90.0	100.3
2013	153	20.0	21.0	23.9	28.7	33.2	76.0	80.0	88.0	99.0	109.3
a) Log 2007/2010	--	0.0	0.0	−0.5	−1.3	−2.0	0.0	0.0	−0.5	0.0	0.1
b) Log 2007/2013	--	0.0	0.0	−0.2	1.7	3.1	3.0	2.8	2.5	4.1	3,8
c) Log 2010/2013	--	0.0	0.0	0.4	3.0	5.1	3.0	2.8	3.1	4.1	3,7
Females											
2007	314	19.0	20.3	22.7	25.6	27.9	64.0	68.0	75.0	85.0	90.0
2010	328	19.0	20.0	22.2	26.0	30.4	64.8	68.0	75.1	86.0	93.3
2013	178	19.0	20.5	23.3	27.0	31.5	69.0	73.0	82.5	93.2	103.1
a) Log 2007/2010	--	0.0	−0.6	−1.0	0.7	3.7	0.5	0.0	0.1	0.5	1.6
b) Log 2007/2013	--	0.0	0.4	1.1	2.3	5.3	3.3	3.1	4.1	4.0	5.9
c) Log 2010/2013	--	0.0	1.1	2.1	1.6	1.5	2.7	3.1	4.1	3.5	4.3

*Log* logarithm, a = 100 log(percentile of 2007 /calculated percentile 2010), b = 100 log(percentile of 2007 /calculated percentile 2013), c = 100 log(percentile of 2010 /calculated percentile 2013)

With regard to the WC in the percentile distributions (p5, p15, p50, p85, and p95), particularly during the 2013 measurement, a positive tendency in increase in central abdominal obesity was observed. However, in the first two years (2007 and 2010), the percentile values were relatively similar but less than those of 2013. This pattern is reflected in the increase in the number of cases of overweight and obesity observed during 2013.

**Fig. 1:**
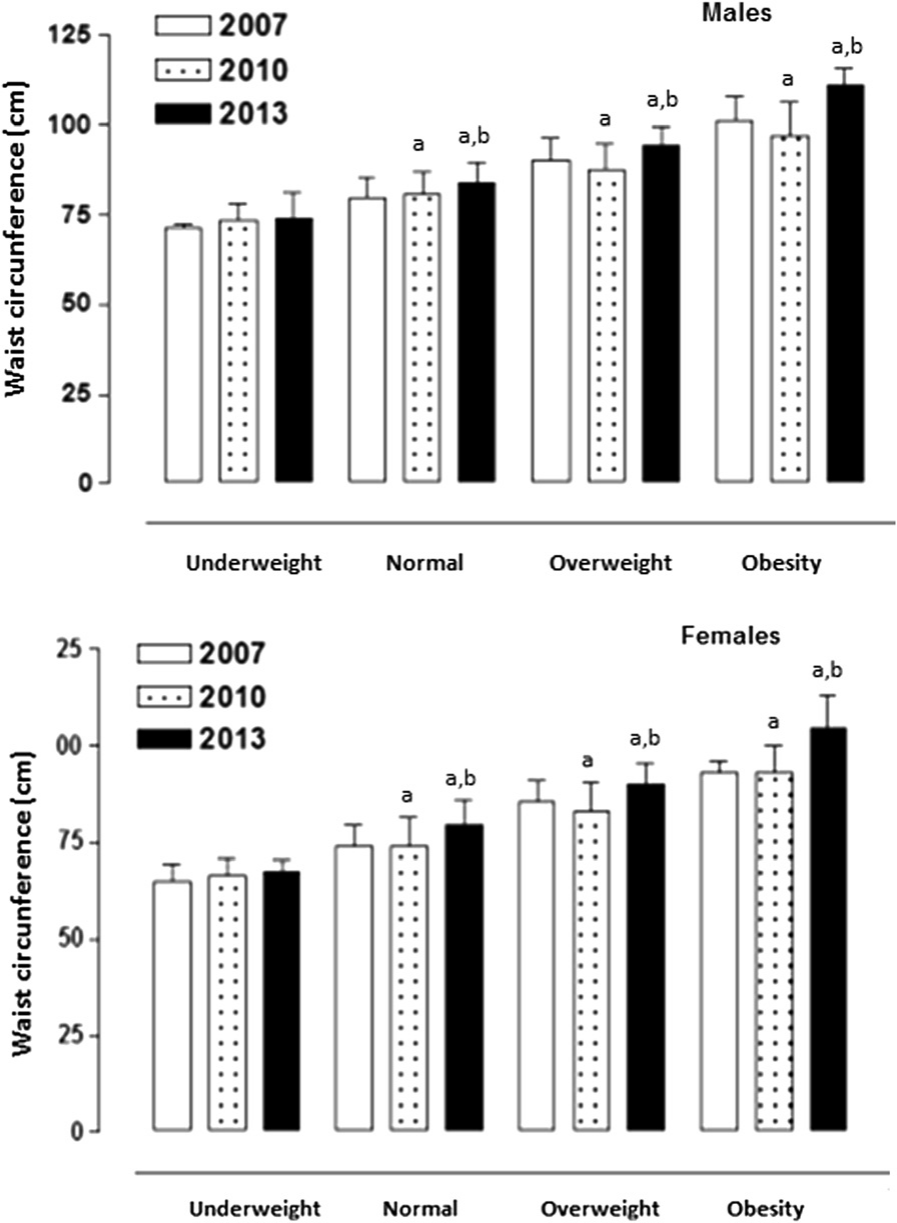
Mean values and ± SD of WC based on BMI categories and measurement years by sex. *a* significant difference in the same nutritional category in 2007; *b* significant difference in the same nutritional category in 2010

Based on the four categories of BMI, Fig. 1 shows the mean and ± SD values for WC. The students of both sexes classified as underweight BMI (less than 18.5 kg/m^2^) showed mean values similar to the WC during the three evaluation years. However, in the other three categories (normal, overweight, and obese), the young university students showed significant increases in WC during 2013 when compared to 2007 and 2010 (*p* < 0.05) where the values remained relatively stable(*p* > 0.05

## Discussion

The results of this study indicate that BMI did not change over time. However, the WC increased significantly in both sexes especially during the last evaluation. The findings reflect the presence of accelerated secular changes in body fat estimated by WC. These findings are consistent with other studies carried out internationally [18–20]. These studies confirmed the increased trends in WC in both sexes extended over time. Since the WC is an anthropometric measure that reflects the central body fat, it is important to point out that the greatest increase in body fat distribution occurred from 2010 to 2013.

These findings are not only worrisome for the sample of university students studied in Talca but also those university students living in the surrounding Maule region of Chile. Therefore, it is important to take preventative measures to control excess weight and obesity in young Chilean university students [10]. Also this research may be used to raise awareness in other countries since recent studies continue to show elevated rates of overweight and obesity in university students around the world [10, 13]. Evidently, this trend can be interpreted as greater levels of abdominal body fat creating a potentially greater risk for young university students to develop related illnesses such as type 2 diabetes, hypertension, dyslipidemia, cardiovascular illnesses, cancer, and death caused by obesity [18, 21, 22].

In general, a large portion of our sample of both males and females in the 2013 evaluation year of this study were found to be above the normal standard values. Young university students included in this study in percentiles 85 and 95 had higher values in the WC. These findings suggest that during very short intervals of time, the body shape of these young university students changed at an accelerated rate.

In essence, our results demonstrate that a large portion of the university population is at significant risk for developing chronic non-communicable diseases. This is due to the greater WC for the same BMI particularly during the 2013 evaluation measurement. In fact, this behavior could be associated with multiple factors such as poor eating habits, an increase in sedentary behaviors [23]. In general, these factors could create the predisposition for the disproportionate increase in WC among the young university students in this sample studied. However, economic development, increased urbanization, and excessive consumption of fats [24] in Chile during the past few years cannot be ruled as contributing factors in these changes observed.

In 1988, the Chilean government created the National Council for Health Promotion and introduced public policies in an attempt to combat the challenges of the epidemiology of obesity [25]. Other endeavors were also undertaken like the proposal for a Global Strategy to Combat Obesity introduced at the 2006 EGO-Chile [26]. Moreover in 2012, approval and publication of the national nutritional labelling law for food were obtained [27]. Despite these government’s efforts, abdominal fat, at least among the young university students studied, continues to increase on a grand scale.

Identifying the limitations in this study will help improve the design for future research projects. First of all, the sample cohorts were selected through non-probabilistic sampling (accidental). This does not allow generalization of the results to other university populations in Chile. Therefore, these findings should be interpreted with caution. In the second place, it was not possible to measure more variables related to body composition, physical activity, and eating habits of the sample. Therefore, in future research, these variables need to be taken into account since controlling for these variables could contribute to identifying the actual factors that contribute to the excessive increase in obesity in young university students. Furthermore, we suggest extending the research to other regions in Chile with the goal of including other universities with similar characteristics.

On the other hand, the process of data collection took place appropriately by maintaining a standardized protocol with an evaluation team with extensive experience in anthropometric measurement. Furthermore, the sample from this project was representative since it included students from the majority of the professional programs. This allowed a greater evaluation of changes in body fat amongst diverse University of Talca Chilean students. Moreover, during the three measurements taken, the same intervals of time were maintained between the evaluations. Furthermore, a strict order and sequencing in the methodology was adhered to ensuring an adequate methodological design.

## Conclusion

In conclusion, the results showed that the BMI remained constant during 2007, 2010, and 2013. However, the young university students of both sexes may have a tendency for a greater risk of abdominal obesity. These results suggest a potential adverse cardio metabolic risk in the university populations of Talca. Thus, it is urgent that obesity prevention programs be instituted within the universities.

## Abbreviations

BMI: Body Mass Index
WC: Waist circumference
SD: Standard deviation
UTAL: University of Talca, Lircay Campus
TME: Technical Measurement Error
